# Muscle Synergies-Based Characterization and Clustering of Poststroke Patients in Reaching Movements

**DOI:** 10.3389/fbioe.2017.00062

**Published:** 2017-10-13

**Authors:** Alessandro Scano, Andrea Chiavenna, Matteo Malosio, Lorenzo Molinari Tosatti, Franco Molteni

**Affiliations:** ^1^Institute of Industrial Technologies and Automation (ITIA), Italian National Research Council (CNR), Milan, Italy; ^2^Rehabilitation Presidium of Valduce Ospedale Villa Beretta, Lecco, Italy

**Keywords:** muscle synergies, centroids, synergies clustering, reaching performance scale, Fugl-Meyer assessment

## Abstract

**Background:**

A deep characterization of neurological patients is a crucial step for a detailed knowledge of the pathology and maximal exploitation and customization of the rehabilitation therapy. The muscle synergies analysis was designed to investigate how muscles coactivate and how their eliciting commands change in time during movement production. Few studies investigated the value of muscle synergies for the characterization of neurological patients before rehabilitation therapies. In this article, the synergy analysis was used to characterize a group of chronic poststroke hemiplegic patients.

**Methods:**

Twenty-two poststroke patients performed a session composed of a sequence of 3D reaching movements. They were assessed through an instrumental assessment, by recording kinematics and electromyography to extract muscle synergies and their activation commands. Patients’ motor synergies were grouped by the means of cluster analysis. Consistency and characterization of each cluster was assessed and clinically profiled by comparison with standard motor assessments.

**Results:**

Motor synergies were successfully extracted on all 22 patients. Five basic clusters were identified as a trade-off between clustering precision and synthesis power, representing: healthy-like activations, two shoulder compensatory strategies, two elbow predominance patterns. Each cluster was provided with a deep characterization and correlation with clinical scales, range of motion, and smoothness.

**Conclusion:**

The clustering of muscle synergies enabled a pretherapy characterization of patients. Such technique may affect several aspects of the therapy: prediction of outcomes, evaluation of the treatments, customization of doses, and therapies.

## Introduction

Neurological diseases are one of the main sources of disability, especially in Western countries. A wide variety of pathologies and symptoms can lead to partial or complete disability, which influences people life up to preventing self-autonomy. A recent state-of-the-art review on rehabilitation (Krebs and Volpe, 2015) concludes with the following sentence: “Ultimately the goal should be determining how best to customize the treatment protocol to meet each individual patient’s needs.” In order to maximize the gains inducted by rehabilitation, patients should undergo a customized treatment protocol tailored on specific criteria that would assure the highest probability of success, depending on their clinical status. For this reason, patients should be evaluated and categorized when possible before the therapy to ensure the clinician with a wide, detailed and deep characterization of the motor capabilities of the patient to be treated. The need of assessing patients’ clustering based on impairment is discussed in the literature (Baldassarre et al., 2016). Such issue is of great interest and still far from being successfully exploited. Many criteria have been proposed to address the subjects to the most suitable approach. Recently Wolf et al. (2016) proposed an algorithm to decide the techniques and tools the physical therapist should choose to provide a high quality treatment to acute stroke patients. The algorithm is based on some key items of the Action Research Arm Test (ARAT) and, based on the scores acquired, the algorithm suggest to the clinician a list of techniques to provide (i.e., passive range of motion [ROM], strength training, motor imagery, mirror therapy). The key items provide a synthetic portrait of patient’s capabilities and a consequent clustering according to its functioning. The decisional method proposed in Wolf et al. (2016) focuses on active movement capacity as predictor of a given outcome. Many other works rely on active movements of clinical scales assessment to estimate patients’ behavioral cluster (Baldassarre et al., 2016) and consequent therapy functional prediction (Kwakkel et al., 2003). For example, patients who are not able to maintain 90° of shoulder flexion can either gain no function to normal function assessed with ARAT (Puig et al., 2011). These approaches generally have good correlation with actual upper limb recovery but suffer of interindividual variability and do not take into account others crucial factors that can be evinced only with instrumental evaluation.

Relatively new branches of rehabilitation, such as robot-aided mobilization, are becoming standard practice in clinical environment. However, even in refined, state-of-the-art studies assessing the effect of rehabilitation (Lo et al., 2010), patients are administered therapies with deep regard related to their characterization before the therapy, which is usually confined to clinical scales scores. Consequently, patients’ characterization is often demanded to clustering related to the severity of impairment (severe, moderate, and mild) depending on clinical scales [such as the Fugl-Meyer Assessment (FMA)]. Such points are assessed in Krebs and Volpe (2015), where many questions are moved about how machines and robots should intervene to help the motor recovery. The answer is implicitly that, so far, no precise answer can be given due to the lack of data and knowledge about patients’ status.

Such observations coming from recent literature suggest that multi-domain approaches, related not only to clinical scales but also to other domains, such as instrumental evaluations, might instead be useful for at least two reasons. First, they might provide deeper assessment; secondly, they might suggest different grouping and, consistently, different characterization. The coupling with instrumental evaluation should become a more detailed procedure that helps in orienting therapies for neurological patients, providing deeper characterization.

Surface electromyography (EMG) has been widely studied on stroke subjects and many alterations have been found compared to healthy individuals. EMG is an interesting assessment since, despite its limitations in terms of cross-talking, electrode placement, repeatability (De Luca et al., 2010), represents measures that are taken “directly on patients.” Relevant results were found in stroke patients EMG patterns, like abnormal coactivation of shoulder and elbow muscles, altered activation pattern, or global decrease of muscle activation (Dewald et al., 1995). s-EMG is used as a technique for clustering patients and healthy people depending on their muscular activations in lower limbs tasks (Miljković et al., 2011).

Moreover, in the past two decades, the concept of muscle synergies was introduced (Flanders and Herrmann, 1992). Muscle synergies are groups of coactivating muscles that, being controlled as a synergic group, allow simplifying the problem of motor control. In fact, the muscle synergies analysis investigates the hypothesis that the central nervous system (CNS) simplifies the problem of motor control by exploiting motor abundance, i.e., the redundancy of actuators in respect to the actuated joints. While the human neuromusculoskeletal system can provide infinite solutions to the motor control problem, the synergies-based approach implies that abundance is not a source of computational burden (Latash, 2012). In fact, neural encoded coactivating synergies drastically reduce the number of requested motor commands. Consequently, a restricted number of synergies is the “basic set of vectors” that represent the invariant element that is shared by a group of motor programs. Furthermore, invariant synergies are modulated by an activation command that specifies the contribution of each synergy to voluntary movement at each time sample. Thus, the synergy approach decomposes the problem of motor control into two less expensive and reusable domains (synergies and their activation commands), to whom the CNS can draw to ease its control over movement. Several approaches and algorithms have been proposed for synergies extraction (Tresch et al., 2006).

A review study analyzed the most exploited methods for synergies extraction, such as principal components analysis (PCA), factor analysis (FA), independent component analysis (ICA), and non-negative matrix factorization (NMF) (Tresch et al., 2006). PCA has been widely adopted (Saltiel et al., 2001; Todorov and Ghahramani, 2004; Tagliabue et al., 2015). However, due to its intrinsic feature of requiring orthogonality between the data set, PCA can lead to negative muscle activation. For these reasons, PCA-based studies may rely on additive methods to constrain coefficients to be positive. FA and ICA are less used and suffer from extraction and rotation issues (Merkle et al., 1998). NMF instead returns always positive synergies compositions and activations, being non-negative by nature. Furthermore, it is a reliable method that reflects the features of EMG signals (d’Avella et al., 2008; Razavian et al., 2015).

Synergies dataset composition is usually compared by matching them according to the similarity of their dot product. Another common method to compare synergies after their extraction is clustering. Clustering was usually conducted with k-means (Steele et al., 2015) or hierarchical clustering (García-Cossio et al., 2014). Such procedures are needed in attempts to provide physiological interpretation of the results (i.e., coupling each synergy with a physiological function within the examined motor task). However, clustering often leads to uneasy clinical interpretation.

Synergies analysis has a twofold applicability. On healthy people, it can be used to investigate motor control, and provide a solid characterization of physiological movement and of the mechanisms that underlie motor production. In particular, the modular organization of our CNS for motor production is tested, suggesting that a reduced number of modules (synergies and their activation profiles) account for the majority of the EMG activity related to specific tasks. Previous studies investigated specific aspects of functional movements, with deep analysis on frontal reaching (d’Avella et al., 2006, 2008), and provided characterization of healthy physiological performance; in Coscia et al. (2014), repeatability and solidity of extracted synergies of healthy people in interaction with devices for rehabilitation during reaching movements was demonstrated.

On neurological patients, synergy analysis may provide further insights and assessment of patients’ clinical condition, providing further characterization related to activation patterns. Previous studies investigated neurological patients’ motor performances in the framework of muscle synergies, with respect to control subjects or to the same patient’s less affected limb. Lunardini et al. (2017) applied synergy analysis on upper limb muscles of children with dystonia and age-matched healthy controls during the performance of different writing tasks. The results suggest that dystonic children should have access to an intact set of synergies with normal structure and that the aberrant muscle activity may result from abnormal recruitment of intact motor modules (Safavynia et al., 2011). Other studies identify alteration of muscle synergies according to three different patterns: preservation, merging and fractionation (Cheung et al., 2012). Preservation is the similarity of muscular synergies between impaired and less impaired arm but with different muscular activation patterns. Merging consists in a reduction of synergies on the more affected limb. Merging was found also in other studies (Clark et al., 2010). Last pattern is fractionation, which consists in the splitting of a synergy in two (or more) synergies. Even fractionation was found on chronic stroke patients. In Cheung et al. (2012), a large number of synergies are required to successfully explain the more affected arm EMG, in respect to healthy people. Further studies did not observe merging nor fractionation patterns in severe patients’ synergies during robot-assisted motion, as the total number of synergies reconstructed was the same of healthy control subjects, but with some altered muscle activation (Roh et al., 2013). A study on patients’ affected by multiple sclerosis, revealed that the number of synergies that underlying gait is comparable to healthy controls, and alterations are observed mainly on activation timing rather than synergies composition (Lencioni et al., 2016).

Such results from the literature can be summarized by underlying that pathological subjects present a wide variety of synergies alteration that are not easily classified. However, their comprehension is of primary importance for better knowledge of the pathology and to provide the best therapy and assistance.

Few studies in the literature tested muscle synergies in the domain of neurological patients (Cheung et al., 2009, 2012; Clark et al., 2010; Roh et al., 2013, 2015; Mcmorland et al., 2015). While on healthy people (d’Avella et al., 2003, 2008; Coscia et al., 2014) usually comparable synergies are extracted (related to low variability in movement execution), neurological patients differ consistently in terms of activations and motor production. The ROM might be altered, as well as smoothness, repeatability, and muscle recruitment timing. These features introduce high variability that lead to: (1) alteration in synergies composition, (2) alteration in timing of synergies elicitation, (3) merging and fractionation issues, and (4) difficulties in determining metrics for synergies comparison.

The analysis conducted in the literature usually focuses on clustering of patients and/or healthy subjects depending on the number of extracted modules (Clark et al., 2010; Roh et al., 2013). However, correspondence among synergies is not easily detectable, especially when neurological patients are involved, since they show a wide variety of motor impairments. For these reasons, in this article, a solid method for clinical interpretation of clustering, in the framework of muscle synergies, is proposed to provide characterization on a cohort of poststroke patients.

### Objective

The aim of the study was to use muscular synergies for a deep characterization of poststroke patients prior to rehabilitation therapies.

## Materials and Methods

### Setting

The study took place at Presidio di Riabilitazione dell’Ospedale Valduce Villa Beretta, Costa Masnaga (LC), Italy, during the period ranging from years 2014 to 2016. The study was reviewed and approved by the local Ethics Committee at Lecco Manzoni Hospital and was conducted in compliance with the Declaration of Helsinki. Written informed consent was obtained from each subject before inclusion in the study.

### Participants

Twenty-two poststroke patients in the chronic phase (more than six months from the stroke event) were recruited for the study. All patients had motor deficits in one upper limb. No requirement on motor functionality was requested, even if patients were selected, with different levels of impairment. Eligibility criteria also included comprehension of the tasks to be performed. The demographic characteristic of the patients are listed in Table 1. Clinical characteristic of the patients are listed in Table 1, including reaching performance scale (RPS) and FMA scores.

**Table 1:** Patients’ demographic and clinical data.

Patient	Gender	Age	Months from stroke	Impaired hand	Type of stroke	Reaching performance scale	Fugl-Meyer Assessment (sections A–D)
Pt1	Male	67	6	Right	Ischemic	17	61
Pt2	Male	65	6	Right	Ischemic	15	56
Pt3	Female	46	168	Right	Ischemic	17	61
Pt4	Male	62	76	Right	Ischemic	12	50
Pt5	Male	49	19	Right	Hemorrhagic	12	36
Pt6	Male	82	8	Right	Ischemic	7	39
Pt7	Male	80	27	Left	Ischemic	9	40
Pt8	Male	74	10	Left	Ischemic	11	56
Pt9	Female	35	44	Right	Hemorrhagic	16	48
Pt10	Male	56	151	Right	Ischemic	9	46
Pt11	Male	66	66	Left	Hemorrhagic	10	48
Pt12	Female	24	32	Right	Ischemic	10	41
Pt13	Male	73	8	Left	Ischemic	10	40
Pt14	Male	30	10	Left	Ischemic	9	33
Pt15	Female	68	51	Right	Ischemic	12	45
Pt16	Female	65	6	Left	Ischemic	6	29
Pt17	Female	26	6	Right	Hemorrhagic	7	22
Pt18	Female	76	27	Right	Ischemic	5	18
Pt19	Male	55	32	Left	Ischemic	1	24
Pt20	Male	65	11	Left	Ischemic	4	22
Pt21	Male	31	44	Left	Ischemic	1	17
Pt22	Male	51	118	Left	Ischemic	1	11
Means	15M-7F	56.6 ± 17.9	42.1 ± 47.3	12R-10L	18I-4H	9.1 ± 4.9	38.3 ± 14.7

### Study Outline

**Figure 1:**
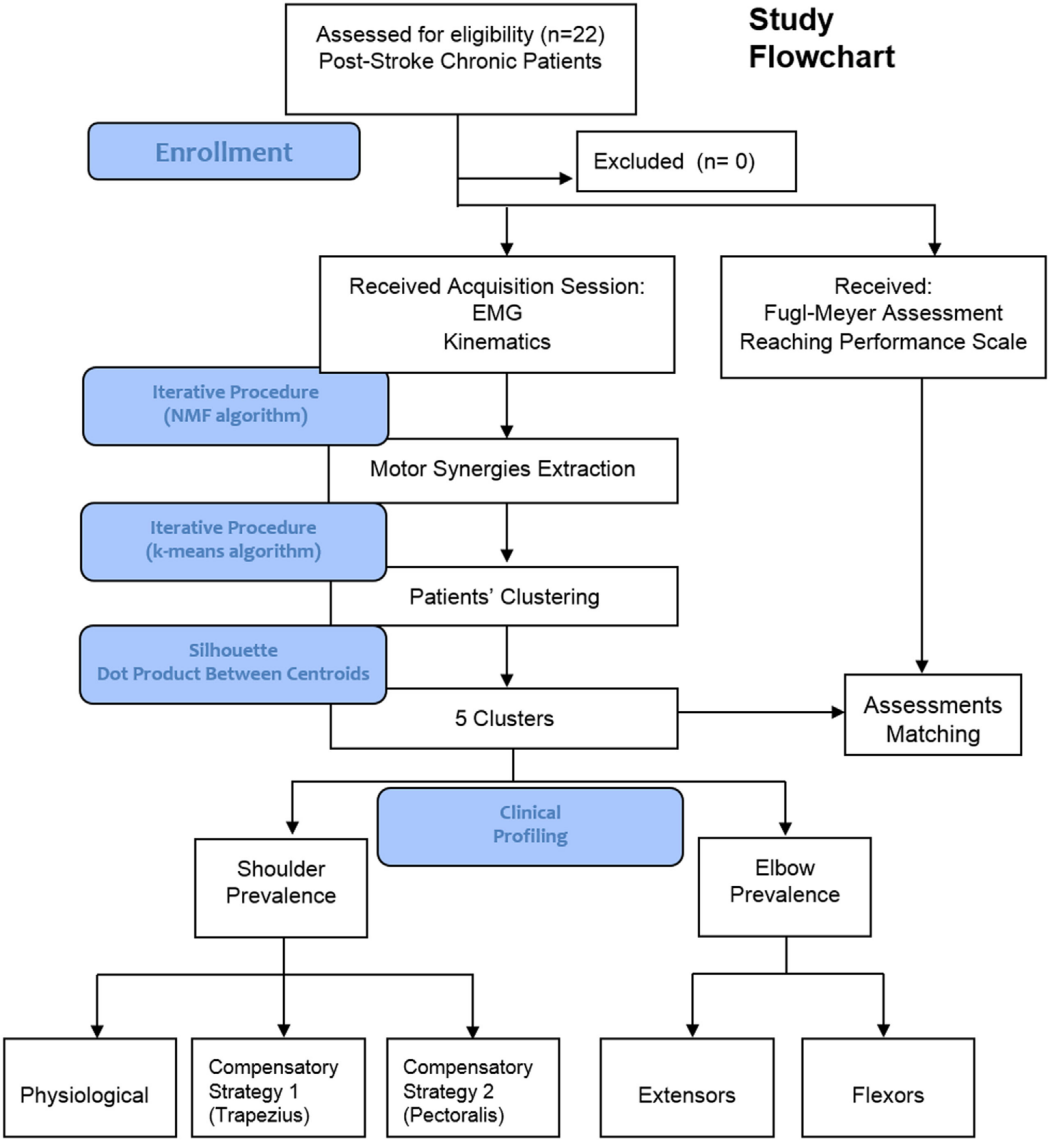
Study flowchart (includes main results).

**Figure 2:**
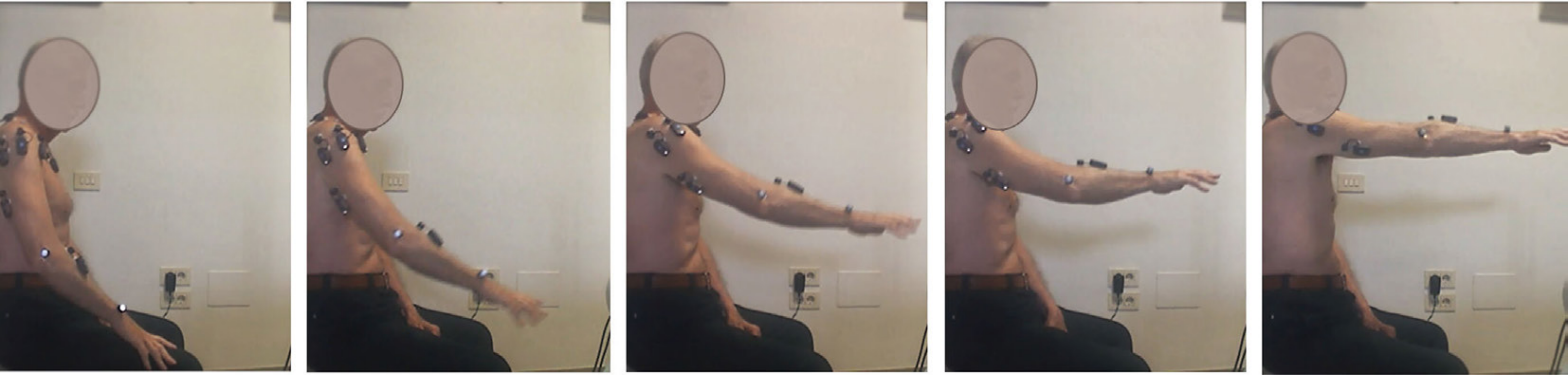
The 3D frontal reaching movement executed by patient 1.

A Flowchart illustrating the outline of the study is shown in Figure 1. Twenty-two patients were recruited and asked to perform several repetitions of reaching movements (Figure 2). None of them was excluded from the study. s-EMG of 8 involved muscles were recorded and used to extract muscle synergies patterns. Synergies were clustered and patients grouped according to their EMG patterns. Each group was provided with a clinical profiling. Clinical scales were used as reference to comment and discuss muscle synergies groups. Main results underlined the emergency of five distinct clusters, which distinguish between shoulder and elbow prevalence patterns. Shoulder patterns split into healthy-like activations, and two compensatory shoulder patterns. Distal patterns distinguish between flexor or extensor predominance. The comparison with clinical scales underlined that synergies clustering does not univocally correlate with standard clinical assessments.

### Equipment

The following equipment was used in this study:

- BTS Smart-D system for kinematics (recorded at 140 Hz) and EMG (recorded at 1,000 Hz);

Kinematics was recorded for D5 and C7 vertebras, acromion, elbow epicondyle, styloid process of the ulna.

s-EMG was recorded on the following Selected Muscles: upper trapezius (Tr), pectoralis major (Pm), deltoid anterior (Da), deltoid middle (Dm), deltoid posterior, triceps brachii caput medialis, biceps brachii caput longus, and brachioradialis (Br).

### Patients Clinical Evaluation: Clinical Scales

Clinical evaluations were performed by a physical therapist using the FMA (Potter et al., 2011) and the RPS (Levin et al., 2004). The FMA is a stroke-specific, performance-based impairment scale, belonging to the body function domain of the ICF model, designed to assess motor functioning, balance, sensation, and joint functioning in patients with poststroke hemiplegia. Specifically, in this study, we used only the upper extremity motor section of the FMA (scale 0–66, 66 = no motor deficits).

As a second assessment scale, The RPS (Levin et al., 2004, 2012) was used. It is a clinical scale that monitors the execution of 3D reaching gestures. It is composed of six sections, monitoring the following characteristics of movement execution: trunk, smoothness, shoulder, elbow, prehension, and global impression. Each section is given a score ranging from 0 to 3 depending on the quality of the performances. In this study, which evaluates the synergies elicited in frontal reaching movements, the RPS is used as comparison to discuss muscular synergies cluster composition.

### Synergies Extraction

First of all, kinematic recordings were used to separate movement phases. Data from retroreflective markers were filtered with a low-pass, third order Butterworth Filter, with cutoff frequency set at 6 Hz. An algorithm for automatic phase-detection was implemented, using wrist vertical coordinate. In this study, only forward phases were considered (involving shoulder flexion in the sagittal plane and elbow extension, simulating a frontal reaching up to shoulder height). Data from eight s-EMG channels were recorded. Data were filtered with a Hilbert transform filter that allows the loss of the minimal amount of signal. EMG data from each subject and each trial were pooled together in a single aggregated matrix and synergies were extracted using the NMF algorithm (Cheung et al., 2005). The NMF decomposes the EMG matrix into the product of two matrices, the first one representing time-invariant, neural coded synergies (*w_i_*), and the second one representing time-variant activation commands for each synergy (*c_i_*) (d’Avella et al., 2006), as in Eq. 1:

(1) $$\text{EMG}(t)=\sum_{i=1}^{N}c_{i}w_{i},$$

where for each of the recorded muscles, EMG(*t*) represents the EMG data at time *t* and *N* is the total number of extracted synergies.

The order of the factorization *r* was chosen increasingly from 1 to 8 (maximum number of muscles that characterizes the dimensionality of the problem). For each *r*, the NMF algorithm was applied 1,000 times in order to avoid local minima and the repetition accounting for the higher variance of the signal was chosen as the representative of order *r*. The number of synergies was chosen as the minimum *r* explaining at least 0.80 of the variance of the signal (Coscia et al., 2014).

### Muscle Synergies in Reaching Movements in the Literature

**Figure 3:**
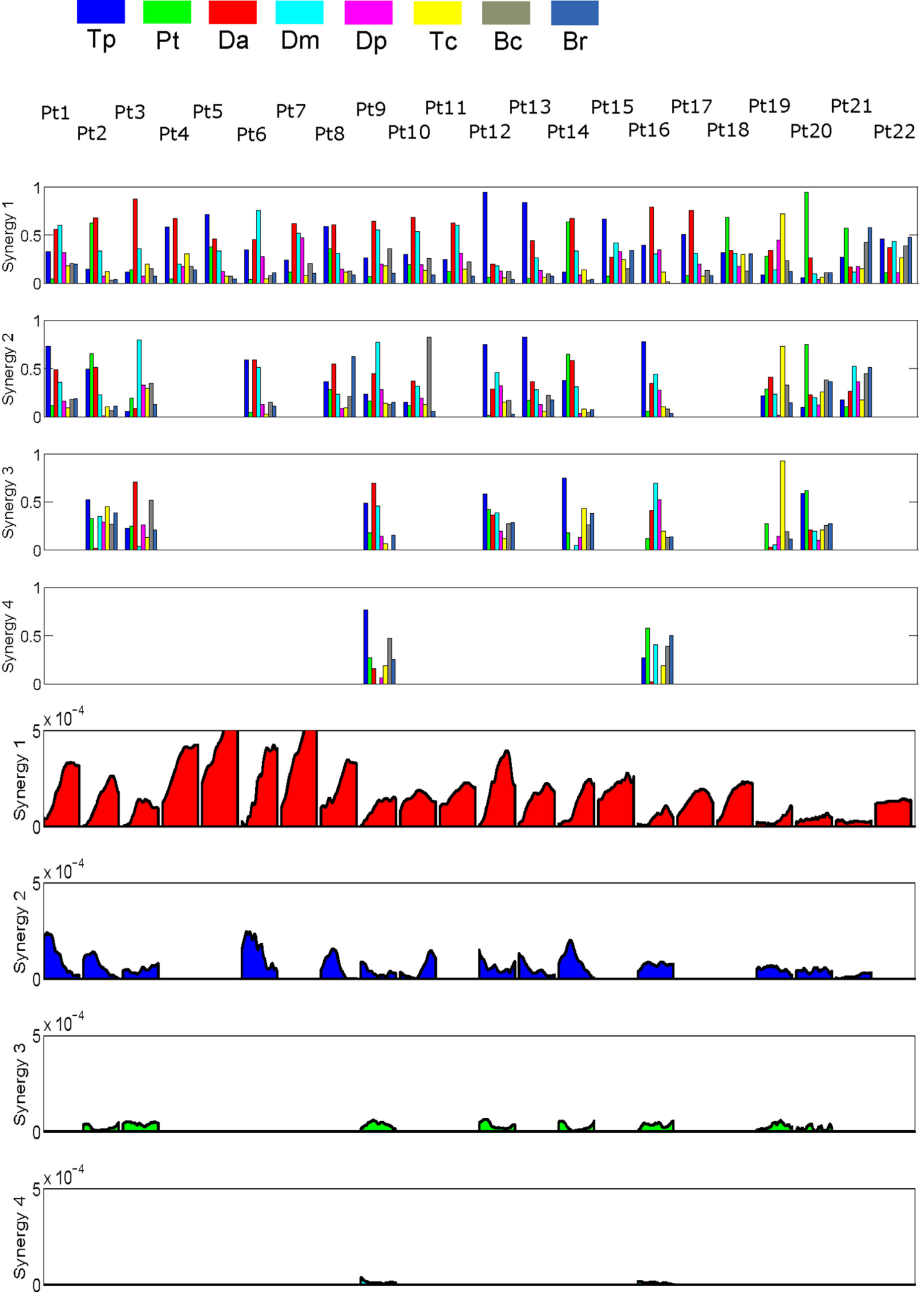
Patients’ extracted synergies. Rows 1–4 show synergies composition. Rows 5–8 show synergies activation profiles corresponding to synergies.

In a previous study on healthy people (Scano et al., 2017.), the pattern portrayed in Figure 3 was found. Two basic synergies are extracted on healthy people free movements. The first one (S1) is the “elevation—extension synergy” that is elicited during the whole movement, increasing its intensity up to about 75% of the movement and partially decreasing in the last part when approaching maximum shoulder flexion. S1 involves mainly Da and Dm (muscles responsible for the elevation of the limb), slightly supported by Tr and pectoralis; a coordinated activation is spotted on the triceps that extends the forearm toward the target. Such finding is supported by Kisiel-sajewicz et al. (2011) that states that Da and triceps are the main agonist muscles in reaching movements. A second synergy (S2) involves mainly the Tr and, less consistently, the Da, pectoralis, and biceps. S2 is active especially at the beginning of the movement and is slightly evoked even at the end. Its role is to stabilize the shoulder, by stiffening it to prepare the elevation of the limb. In the end, S2 slightly intervenes to stabilize the limb at the end of the movement and to keep the arm elevated at about 90°, over which the Tr becomes a shoulder elevator.

It is very interesting to compare the synergies extracted on healthy people with the ones found in the literature by other authors. In d’Avella et al. (2006, 2008) motor synergies are extracted while investigating the spatiotemporal features of physiological frontal reaching movements. Five synergies were extracted, starting from 17 muscles EMG. Synergies are ordered by temporal criteria, in chronological elicitation order. The first synergy is dominated by flexors muscles of the elbow and by the Tr. Such a synergy is a stabilizing synergy that prepares the shoulder to the elevation. It resembles clearly the second synergy that was found in Scano et al. (2017). The second, third, and fourth synergies found in d’Avella et al. (2006) are instead related, respectively, to elbow extension, shoulder flexion, and a complement to the previous two functions, respectively. In the presented study, a merging effect was observed, indicating clearly the coupling of shoulder flexion and elbow extension. The coupling was probably observed due to the fact that shoulder and elbow move with similar timing and the algorithm, applied to fewer muscles in the present study, coupled them naturally. S1 in Scano et al. (2017) is thus correspondent to the second and third synergies found in d’Avella et al. (2006). The fourth and fifth synergies found in d’Avella et al. (2006) include muscles that were not included in the present study and of minor relevance in reaching production. Authors conclude previous studies in the literature find that the basic elements of the 3D reaching movements are three: a stabilizing initial effect, followed by a coupled action of shoulder flexion and elbow extension. Authors will hereby refer to the shoulder flexion/elbow extension synergy by “S1.”

### Patients Clustering

Some state-of-the-art articles worked on the effect of therapies on muscular synergies, or in the differentiation between the more affected limb and the less affected one (Cheung et al., 2009; Roh et al., 2015), defining clusters to group synergies according to their composition. All the dataset of the extracted synergies are clustered into a limited number of groups, and changes in cluster numbers and composition are metrics to evaluate the difference between limbs performance, or between groups of patients with different level of impairment.

However, for the purpose of this work, including all the extracted synergies into a single cluster analysis might lead to hard clinical interpretation of the clusters (and patients’ classification) for at least two reasons. First, many studies (Roh et al., 2013) report how synergies related to the same motor function (e.g., shoulder flexion) “split into two or more clusters” (Roh et al., 2015). Such cluster composition makes correspondence between clusters and motor functions not easy interpretable.

Furthermore, muscular synergies are often grouped in the literature regardless of their activation timing. Such procedure might be risky in terms of interpretation since similar activation patterns, related to different functions, might be grouped together. While this issue is less likely to happen on healthy people, the variability in patients is high and might lead to misinterpretation.

**Figure 4:**
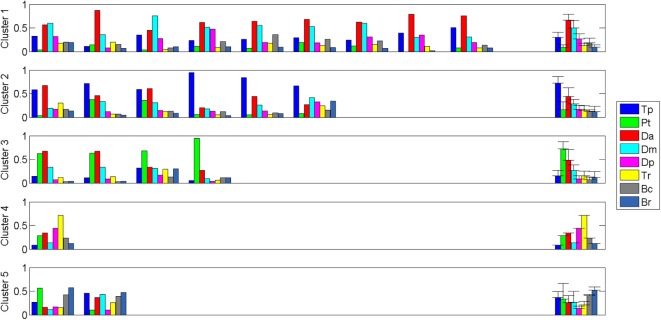
Clusters composition: the S1 of each patient is displayed, grouped in its corresponding centroid.

Such considerations lead the authors to the choice of conducting the cluster analysis considering only S1, since it strongly characterizes frontal reaching movements, as deeply explained in the previous paragraph. Consequently, only synergies sharing similar activation timing (first row for synergies and fifth row for activations in Figure 4) are considered for cluster analysis. Such a procedure is limited to a restricted part of the dataset (the most important one for performing frontal reaching movements) but guarantees that the clustering refers to synergies that perform (or attempt to perform) the same “motor function.”

It should be noted that, in the majority of the cases, the synergy that prevails in terms of entity of activation is also active in the moment of maximum limb elevation, increasing in the last part of the reaching movement. For such cases, the identification of S1 is trivial. However, a very limited number of patients, typically the severely impaired ones, or patients that present a “sparse” group of synergies, may show patterns of more complex identification. This may happen because S1 is partially absent (due to limited ROM) or because it fractionates into two or more synergies. In such cases, the choice of S1 was qualitatively driven by the following criteria: (1) entity of the activation (preferred on severely impaired patients who have limited ROM) and (2) timing of elicitation of the synergy (preferred for patients who present fractionation issues). To authors’ opinion, while still being improvable, this procedure allows a coherent method for the profiling of the clinical status of patients in the framework of muscle synergies.

The cluster analysis was conducted by the means of the *k*-means Matlab cluster algorithm. The algorithm was applied to an aggregated matrix containing the flexion synergy of each patient, selected according to the criteria discussed in the previous paragraph. Each possible clustering order, ranging from 2 (minimum) to 22 (maximum), was tested, by repeating 200 times the algorithm and selecting, for each order, the solution with higher Silhouette Score (García-Cossio et al., 2014).

Then, the selection of the appropriate number of clusters was made by pondering the following metrics:

(1) Parsimonious number of clusters for synthesis power (lowest possible number of clusters, given reasonable descriptive precision).
(2) Silhouette Score, indicating the goodness of the clustering, as a synthetic index for each clustering order. The higher the Silhouette, the better elements fit to their cluster.
(3) Mean scalar product among all the clusters for each cluster order. The lowest the mean dot product, the more cluster are differentiated. Too similar clusters might be grouped together decreasing the order of the clustering.
(4) If possible, single-patient cluster solutions are avoided or at least limited.

Being metrics (1), (2), and (3) not directly comparable, authors decided to select the lowest number of clusters according to whom both the Silhouette Score and the Mean Scalar Product would increase and decrease, respectively. If both conditions are met, increasing the number of clusters has certainly lead to better clustering. Otherwise, increasing the number of clusters may not be needed.

### Matching Clustering with Standard Clinical Assessments and Kinematics

Lastly, extracted clusters are compared to FMA and RPS assessment for detecting correlations between the EMG-based analysis and standard assessments for discussion and further characterization of patients. Furthermore, a comparison with basic ROM assessment was performed. Shoulder flexion angle in the sagittal plane and the elbow extension angle, both measured at maximum shoulder flexion—end of the reaching movement (Scano et al., 2014), were computed. A shoulder flexion of 0° indicates that the arm is leaning along the body; 90° indicates that the arm is fully frontally elevated. An elbow extension of 0° indicates a virtually completely extended elbow. However, due to anatomical differences and marker positioning, a fully extended elbow ranges around the performance of Pt1 (who has maximum elbow scores in both FMA and RPS). As a secondary kinematic-related assessment, authors computed movement smoothness, measured with the Normalized Jerk (Teulings et al., 1997).

## Results

### Extracted Synergies

Extracted synergies and activation profiles are reported in Figure 3. Synergies were ordered, by matching at best activation profiles timing.

### Clustering

Table 2 shows details of Patients clustering for optimal solutions for number of clusters ranging from 2 to 11, according to the criteria explained in the Section “Materials and Methods.” Number of extracted clusters is reported accompanied by Silhouette score, mean dot product between Centroids, and presence of single-patients clusters.

**Table 2:** Clustering details.

Clustering order	Silhouette score	Mean dot product	Single-patient clusters
2	0.50	0.74 ± 0.086	No
3	0.44	0.74 ± 0.081	No
4	0.49	0.73 ± 0.080	No
5	0.54	0.68 ± 0.067	1
6	0.52	0.68 ± 0.065	2
7	0.55	0.67 ± 0.062	3
8	0.61	0.68 ± 0.063	3
9	0.63	0.67 ± 0.065	4
10	0.67	0.68 ± 0.059	4
11	0.71	0.69 ± 0.063	5

*For each clustering order, the optimal solution found with Matlab k-means function is reported, with the Silhouette score, the mean dot product with other clusters, and the number of single-patient clusters*.

Following criterion (1) proposed in the paragraph 2.7, a maximum of 11 clusters was considered, corresponding to 1/2 of the sample. This way, there is the possibility that every centroid is populated by at least two patients. Increasing further the number of clusters would prevent any kind of generalization, and consistently, were *a priori* discarded. In respect to the clustering solution of order 4, the clustering solution of order 5 shows improving of both Silhouette Score and decrease of Mean Dot Product. Consequently, the choice of five clusters was done as a trade-off between parsimony and adequateness of precision in patients’ description. Solutions with 6 or more clusters were all characterized by more than one centroid dedicated to a single subject. Solution with number of clusters = 5 instead had only one single-patient cluster.

**Figure 5:**
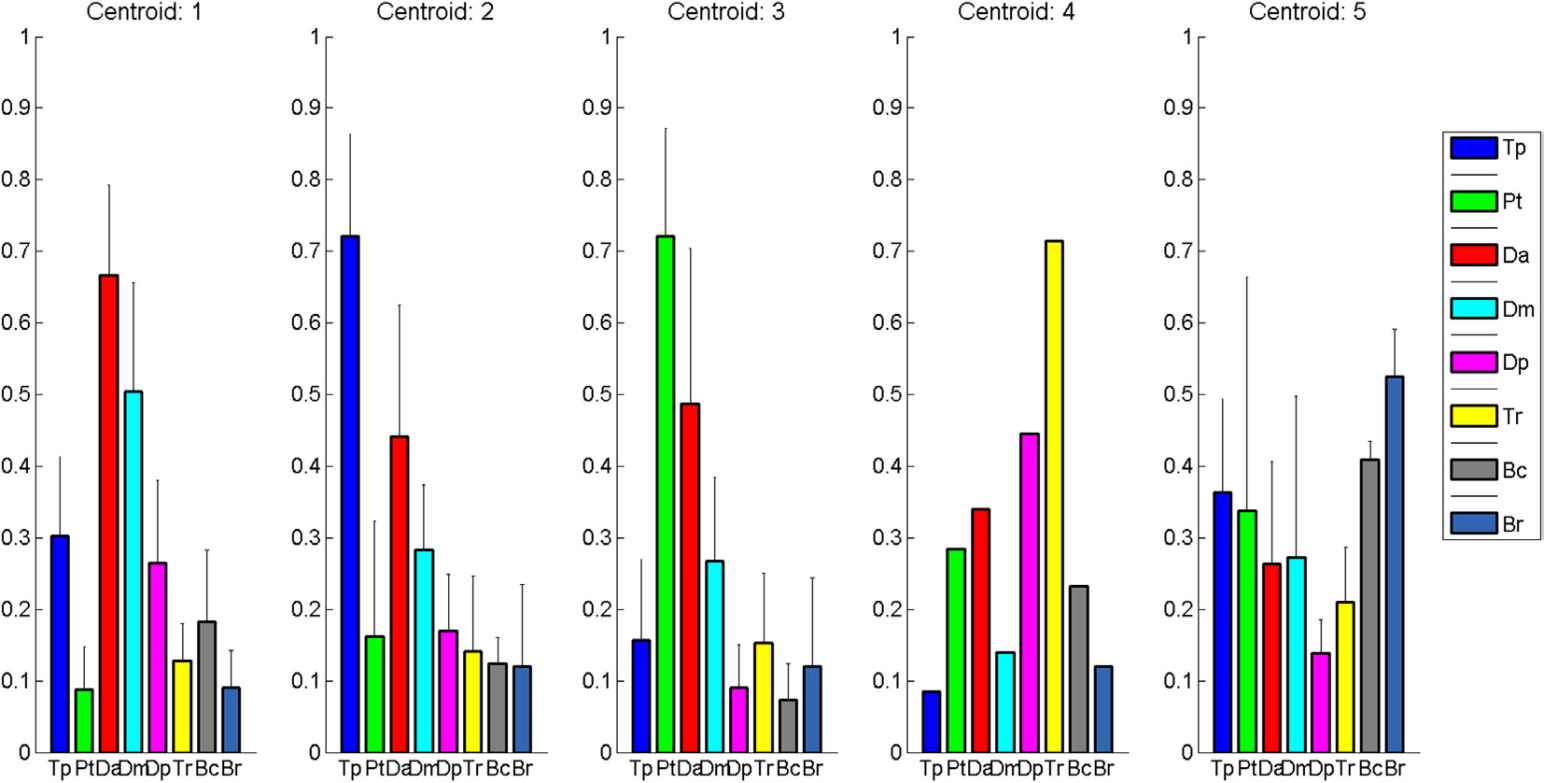
Cluster Centroids extracted with Matlab k-means. Five clusters were identified: healthy-like activations, two shoulder compensatory strategies (based on trapezius and pectoralis), and two distal patterns (one with prevalence of elbow flexors, one with prevalence of elbow extensors).

Clustering Composition for clustering order = 5 is reported in Figure 4 along with the correspondent centroids. Clusters centroids for five clusters grouping are reported in detail in Figure 5 and patients composing each clusters are listed in Table 3.

**Table 3:** Clusters composition.

Cluster	Patients’ ID
1	Pt1, Pt3, Pt6, Pt7, Pt9, Pt10, Pt11, Pt17, Pt16
2	Pt4, Pt5, Pt8, Pt12, Pt13, Pt15
3	Pt2, Pt14, Pt18, Pt20
4	Pt19
5	Pt21, Pt22

*Each cluster is reported with the patients who belong to it*.

Table 4 reports mean dot product between clusters, to assess in detail their similarity.

**Table 4:** Clusters similarity.

Cluster ID	1	2	3	4	5
1	1	0.84	0.70	0.63	0.70
2	0.84	1	0.64	0.54	0.75
3	0.70	0.64	1	0.63	0.71
4	0.63	0.54	0.63	1	0.66
5	0.70	0.75	0.71	0.66	1
Mean	0.72 ± 0.08	0.70 ± 0.13	0.67 ± 0.04	0.62 ± 0.05	0.71 ± 0.04

*Pairwise dot products among clusters are reported along with the “average similarity” of each cluster to the others. Mean dot products do not include the diagonal unitary elements of the matrix in Table 4*.

### Clustering vs. Clinical Scales

**Figure 6:**
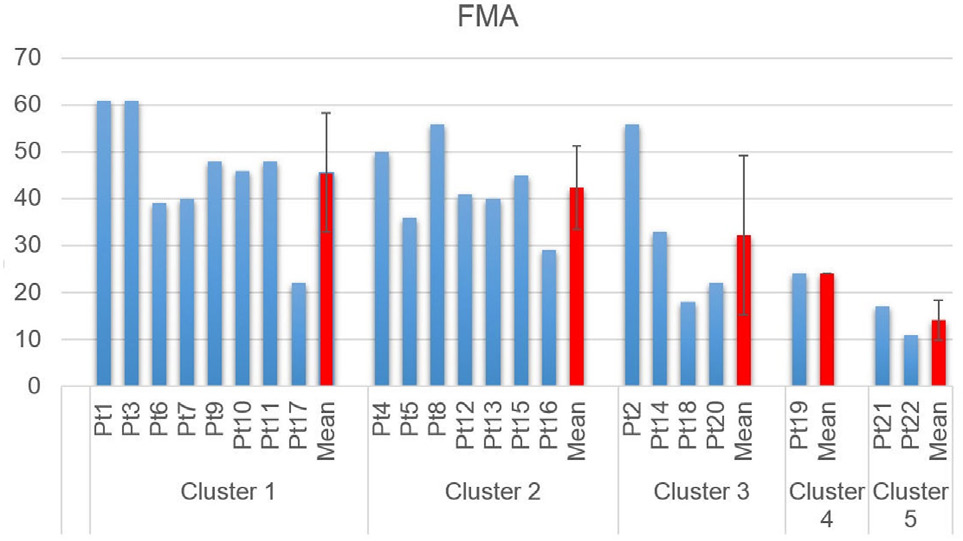
Fugl-Meyer assessment scores, grouped by cluster.

Detailed FMA scores for each of the five clusters is reported in Figure 6, along with mean and standard deviation of each cluster.

**Figure 7:**
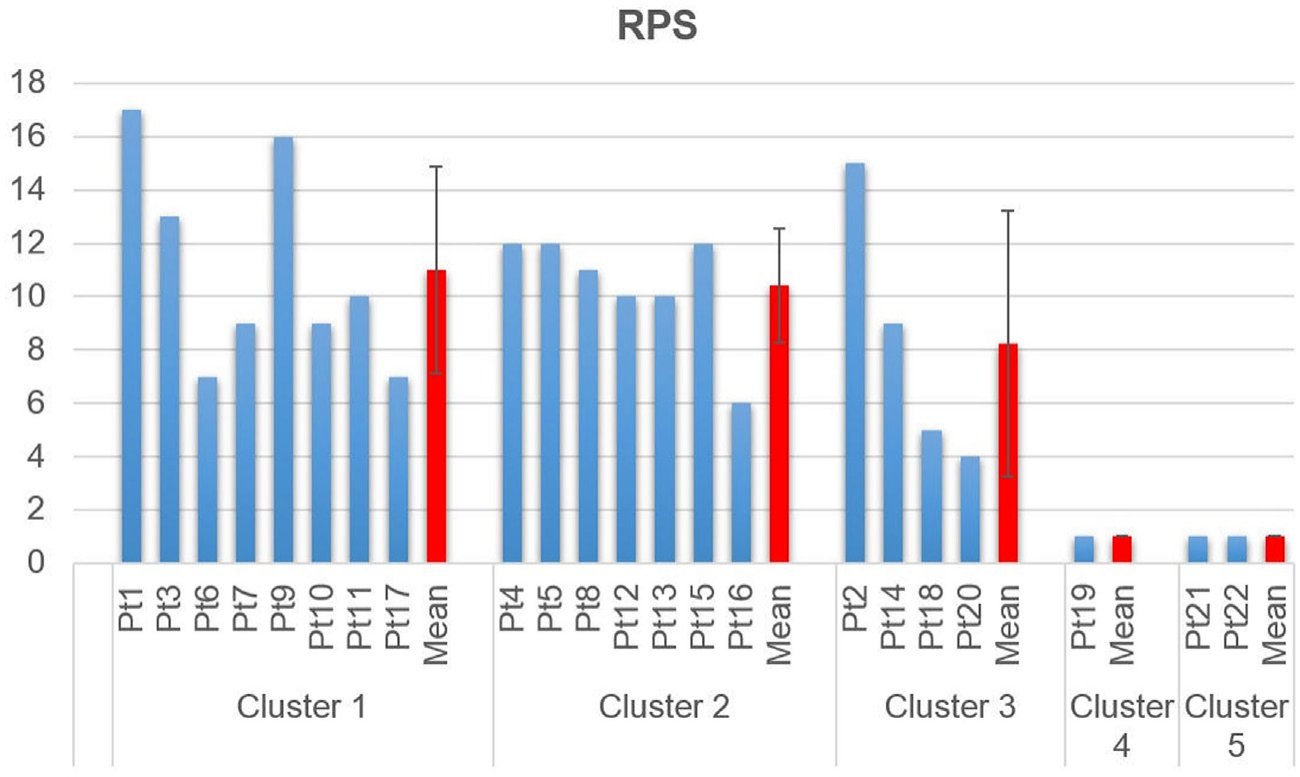
Reaching performance scale scores, grouped by cluster.

Detailed RPS scores for each of the five clusters is reported in Figure 7, along with mean and standard deviation of each cluster.

### Clustering vs. Kinematics

**Figure 8:**
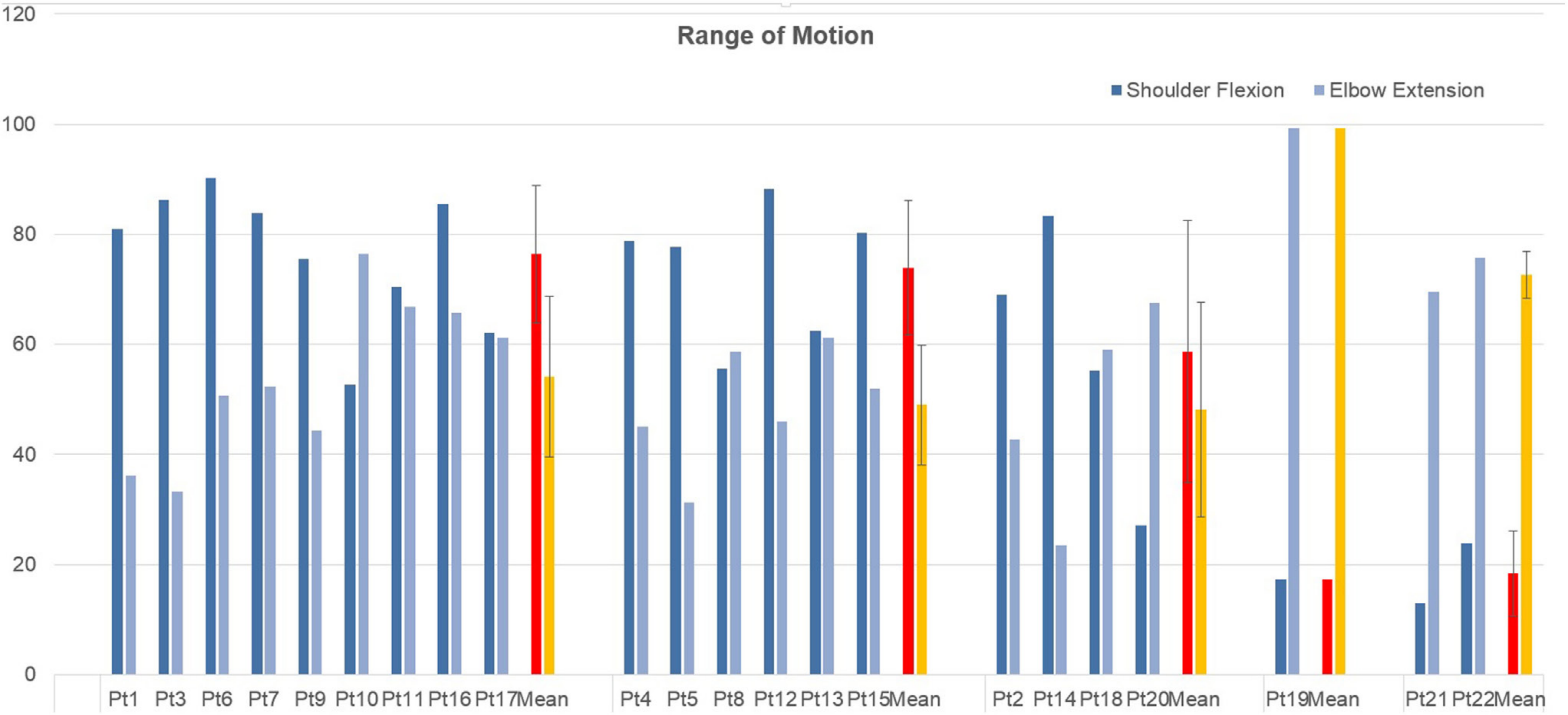
Shoulder flexion angle in the sagittal plane and elbow extension angle, measured at maximum shoulder flexion, grouped by cluster.

Table 5 reports mean shoulder flexion angles, elbow extension angles, and normalized jerk for each patient. Detailed shoulder flexion angle (computed in the sagittal plane) and elbow extension angle, for each of the five clusters is reported in Figure 8, along with mean and standard deviation of each cluster.

**Table 5:** Kinematics.

Patient ID	Shoulder elevation	Elbow extension	Normalized Jerk
Pt1	81.0° ± 1.4°	36.1° ± 5.9°	36.3 ± 11.0
Pt2	69°0.0 ± 2.7°	42.8° ± 2.8°	21.6 ± 7.6
Pt3	86.3° ± 8.0°	33.2° ± 15.9°	60.2 ± 25.5
Pt4	78.9° ± 2.7°	45.1° ± 2.9°	22.2 ± 9.8
Pt5	77.7° ± 1.2°	31.2° ± 1.1°	41.7 ± 8.6
Pt6	90.2° ± 3.5°	50.7° ± 2.7°	59.1 ± 21.0
Pt7	83.9° ± 45.0°	52.3° ± 31.1°	66.2 ± 24.2
Pt8	55.6° ± 5.6°	58.6° ± 4.0°	25.7 ± 9.4
Pt9	75.6° ± 2.3°	44.4° ± 3.0°	32.2 ± 8.5
Pt10	52.6° ± 35.5°	76.4° ± 41.8°	19 ± 5.6
Pt11	70.4° ± 2.6°	66.9° ± 5.6°	49.4 ± 16.6
Pt12	88.2° ± 2.0°	45.9° ± 1.9°	27.4 ± 12.7
Pt13	62.5° ± 24.1°	61.2° ± 9.5°	33.6 ± 31.9
Pt14	83.3° ± 1.4°	23.4° ± 4.0°	30.8 ± 11.7
Pt15	80.2° ± 5.4°	51.9° ± 7.4°	114.9 ± 53.1
Pt16	85.5° ± 4.6°	65.7° ± 4.7°	30.0 ± 32.1
Pt17	62.2° ± 9.1°	61.3° ± 11.4°	49.2 ± 28.9
Pt18	55.3° ± 10.8°	59.0° ± 4.5°	27.8 ± 12.5
Pt19	17.4° ± 11.3°	99.3° ± 2.3°	66.2 ± 44.4
Pt20	27.2° ± 35.7°	67.5° ± 12.2°	81.4 ± 41.0
Pt21	13.0° ± 9.2°	69.6° ± 7.7°	99.6 ± 91.4
Pt22	23.8° ± 11.0°	75.7° ± 12.3°	103.2 ± 59.9

*Each patient is characterized in terms of ROM (shoulder elevation angle and elbow extension, both measured at the moment of maximum elevation of the limb at the end of the forward phase) and smoothness (Normalized Jerk)*.

**Figure 9:**
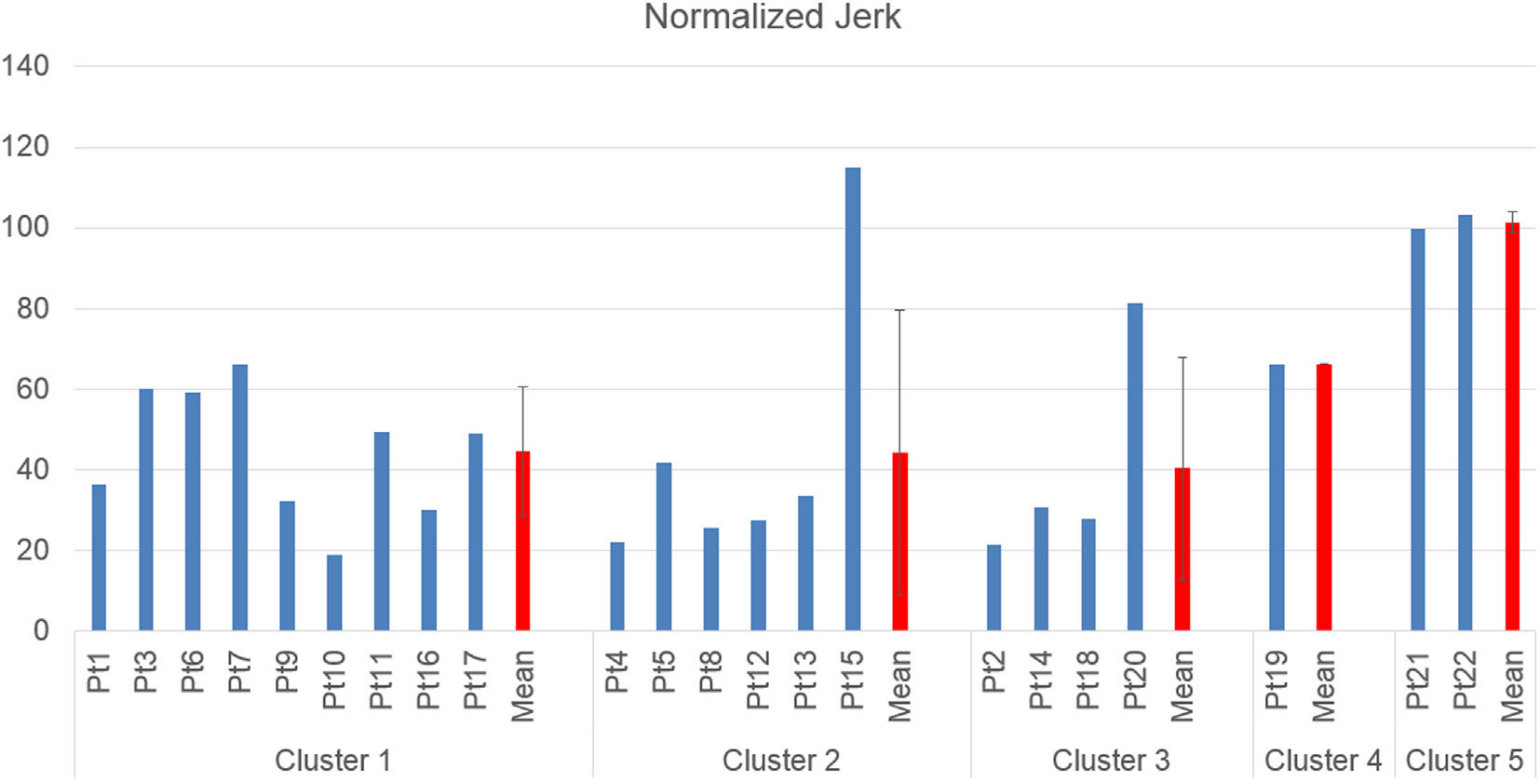
Normalized Jerk, as measure of movement smoothness, grouped by cluster.

Detailed Normalized Jerk scores for each of the five clusters is reported in Figure 9, along with mean and standard deviation of each cluster.

## Discussion

In this article, muscle synergies were extracted and then clustered from a cohort of chronic stroke patients to identify motor patterns for the execution of 3D reaching movements. The muscle synergies method was selected to understand how to characterize the pathological movement and link it, if possible, to the clinical status expressed by clinical scales and kinematics. Compared to less sophisticated EMG analysis, muscle synergies method provides more information about the relationship existing between the muscles, either for what concerns the amplitude and cocontractions, either for the timing of activation that can lead to different interpretation about the role of the muscle during the movement execution (e.g., agonist, antagonist, and stabilizer). All these features are considered in coupling by synergies extraction, adding valuable analysis in respect to single EMG stream analysis. Furthermore, the synergies analysis provides an analytical framework which is coherent with the theories of motor learning and production related to the modular organization of the brain (Schmidt, 1975), and to the vision of motor abundance (muscle redundancy) as a source of simplification of motor control, rather than the opposite (Latash, 2012).

### Consistency of the Extracted Synergies

As for other matrix factorization methods reported in the literature (Tresch et al., 2006; Lin and Scott, 2012; Naik and Nguyen, 2015), the NMF algorithm finds the best decomposition for explaining the major amount of the variance of the original EMG envelope. It means that the method is applied to EMG decomposition without specific knowledge of the nature of the EMG signal itself; the procedure is purely mathematical, and the results reflect such characteristic. It is indeed relevant to investigate not only patterns of coactivating muscles (synergies) but also how repeatable the activation commands are. Such issue is sometimes ignored in the literature. However, if synergies are not stable, their extraction is nothing but the result of a mathematical optimization, without representing real repeatable patterns. This is a fundamental feature of skilled and purposeful motor control, and a requisite to consider synergies extraction as a valuable procedure, rather than just a mathematical procedure. For these reasons, authors considered of crucial importance to apply qualitative selection of the modules to be matched on the basis of the composition of the synergies and of the timing of their activation, introducing critical clinical interpretation of the data. Such point is crucial for a correct synergies matching, even though it is partially limiting since algorithms are applied only to the most relevant synergy for each patient.

### Cluster Extraction

A discussion about the choice of analyzing only elevation synergy is presented. As shown by previous studies found in the literature, it is the main coactivation pattern characterizing the execution of frontal reaching movements. Furthermore, in respect to healthy people, high variability is detected in patients’ patterns (Figure 4), especially considering that in the present study the sample is not homogenous in terms of motor functionality (RPS ranging from 1/18 to 17/18 and FMA ranging from 11/66 to 61/66). Furthermore, as depicted in Figure 4, Synergy 1 not only characterizes the execution of 3D reaching movements, but also includes a major part of the EMG activity. A global clustering, involving all synergies, would equally weight Synergies 2–4 that are less relevant. Extending the analysis to all the extracted synergies, would likely lead to complete impossibility of detecting reliable patterns into the data, due to the absence of matching temporal activation profiles. Matching elevation synergies according to activation profile during the elevation phase was considered as the best way for patients clustering and characterization.

### Cluster Description

Clusters are hereby described according to their clinical profiling.

#### Cluster 1: Physiological Flexion Pattern (Deltoids)

Cluster 1 includes patients that show coactivation patterns that are similar to the ones of healthy subjects (d’Avella et al., 2006; Scano et al., 2017). Overall, Cluster 1 shows the highest mean scores both in RPS and FMA. However, it should be noted that, while high functioning patients are, as expected, grouped in Cluster 1, this group appears as less homogeneous than Cluster 2. In fact, Cluster 1 includes also low-functioning patients such as Pt17. The phenomenon of the joining of high and low functioning patients can be described by the fact that, while motor control features indicate correct activation patterns, low functioning patients are characterized by global weakness that prevents them from compensating with other muscles. It should be noted that, presenting a healthy-like activation pattern, and not being synergies amplitude of activation comparable in terms of magnitude from patient to patient, even higher order clustering would lead to common grouping. Consequently, healthy-like activation pattern (Da dominance, with contribution of Dm) is not a guarantee of high functioning. However, patients belonging to Cluster 1 have intact, selective motor control capabilities and are candidate to motor improvement (low-functioning patients) or motor refinement (high-functioning patients). Kinematics confirm previous considerations. All patients belonging to Cluster 1 have full or nearly full ROM at the shoulder, while some of them do not extend correctly the elbow. The mean Normalized Jerk within the cluster is quite low, indicating a quite smooth movement execution.

#### Cluster 2: Compensatory Strategy 1—(Tr)

Cluster 2 is characterized by a remarkable activation of the Tr muscle, helped by the deltoids. Such patients are in general capable of performing the movement and present complete or quasicomplete ROM at shoulder and elbow. Their pathological condition is explained by a motor control deficit related to the capability of selecting the main agonist muscle (Da), which is replaced by the Tr. Such a pattern, found in chronic patients, might be addressed to the capability of compensating the lack of Da activation to perform the movement. Typical effect of such group is scapular elevation. Cluster 2 groups moderate impaired patients that rely on compensatory strategies. With the exception of Patient 16, this cluster appears to be quite homogeneous both with respect to the RPS and the FMA. The majority of the patients belonging to Cluster 2 have full or nearly full ROM at the shoulder, even if two of them do not complete the elevation of the arm. All of them do not extend completely the elbow. The mean Normalized Jerk within the cluster is low, indicating a quite smooth movement execution, probably to due simplified control related to the uncontrolled, fast elevation relying mainly on Tr. Pt15, suffering of dystonia, represents an expected exception. Cluster 2 appears to have high similarity to Cluster 1 (as reported in Table 4). This result confirms that Cluster 1 and Cluster 2 gather the majority of patients that have better motor performance.

#### Cluster 3: Compensatory Strategy 2—(Pm)

Cluster 3 shows patients who strongly compensate by activating the Pm muscle. Their ROM is often not complete (with the exception of Patient 4) and the compensation may lead to shoulder adduction and intrarotation. Contrarily to previous clusters, the functional level presents high variability but, overall, is consistently lower than the one of previous clusters, as shown by clinical scales. Prognostic indications coming from the belonging to this cluster might be worse if compared to Clusters 1 and 2; main elevator agonists in the sagittal plane play less relevant role than the pectoralis that is not an elevator. However, it should be noted that the composition of this cluster is not homogeneous in terms of clinical scales. Such finding is confirmed by kinematic parameters that range from very good ROM and smoothness (Pt14) to very low performances (Pt20). Cluster 3 is probably the less uniform one.

#### Cluster 4: Distal Prevalence—(Extensors)

Cluster 4 is a single-patient cluster having low proximal functionality, characterized by the prevalence of elbow/distal muscles in the elevation phase. The patient present very low RPS and FMA scores, and have very limited/null ROM in respect to rest/equilibrium poses. The patient belonging to this cluster shows prevalence of triceps activity and lacks muscular tone needed to perform the movement. He is likely to show reduced recovery; kinematics indexes indicate very low ROM and smoothness.

#### Cluster 5: Distal Prevalence—(Flexors)

Cluster 5 is a two-patient cluster. Patients belonging to Cluster 5 show low proximal functionality, characterized by the prevalence of elbow/distal muscles in the elevation phase. Such patient present extremely low RPS and FMA scores and have very limited/null ROM in respect to rest/equilibrium poses. Patients belonging to this cluster show prevalence of elbow flexors muscles (biceps and Br) activity and lacks muscular tone needed to perform the movement. They are likely to show poor prognostic outcomes, since kinematics shows extremely reduced ROM and low smoothness.

### Do these Cluster Represent Poststroke Population?

It might be claimed that the sample of patients in this study splits into two main groups: patients with shoulder prevalence and elbow prevalence. Each group further splits depending on the compensatory strategy used for shoulder flexion (no pattern compensation; Tr compensation; pectoralis compensation) and elbow prevalence (flexors and extensors). This data clustering might be a valuable starting point for interpreting patients’ performance and motor recovery.

Abnormal shoulder abduction and internal rotation during forward (sagittal plane) reaching movements performed by stroke survivors (McCrea et al., 2005) have been interpreted as compensatory responses to saturation of anterior deltoid activation, shifting antigravity support of the arm to other muscles (e.g., medial deltoid). The severity of motor impairment was correlated with the compensatory response (Roh et al., 2013). Compensatory movements can occur *via* the recruitment of additional agonist muscles, thereby distributing the muscle force on other agonists (McCrea et al., 2005). Previous studies showed that in patients lacking the activity of the Pm muscle, have an increased activation of Tr as compensation (Bastlová et al., 2014). The findings of the present study seem to integrate this statement suggesting that patients lacking Da and/or Tr activity might partially compensate with pectoralis, as in Cluster 3, even if with lower motor outcome. Such features are in this study represented by healthy-like flexors (Cluster 1, which does not show pattern alterations in respect to physiological movement), and by two main shoulder compensatory strategies, assigned mainly to Tr (Cluster 2) and Pm (Cluster 3).

Even if very low in number, this study shows two clusters for patients who present mainly EMG activity on the elbow. Cluster 4 is represented by a patient who shows mainly elbow extensor activity, while Cluster 5 groups prevalence on elbow flexion activity. While individuating such difference, both the groups are characterized by global shoulder weakness and poor motor outcomes in 3D reaching movement (null or very low shoulder flexion, low clinical scales scores).

As a global remark, and main result of the study, authors observed that muscular synergies profiling does not match precisely the evaluation provided by clinical scales, suggesting that the evaluation provided by standard tools should be integrated for complete assessment and patients characterization. Consequently, clinical scales might be insufficient for correct and deep patients’ profiling and therapy customization.

### The Trade-Off between Clustering Accuracy and Synthesis

Authors observed that, starting from quite low-order clusters (5) algorithms for clustering extraction tend to create single-patient centroids (at least in the group of patients examined in this study). Order 7 clustering individuates three single-patient-based centroids. Such findings denote the difficultness in grouping patients that are in general characterized by their own peculiar muscular patterns. Consequently, cluster analysis suggests that patients tend to show individual patterns. However, order 5 clustering was considered as a reasonable grouping order to provide deep enough characterization. It detects a centroid characterized by healthy-like activations, two major compensatory strategies, and two severely impaired groups.

### Muscle Synergies Clustering as Part of Multidomain Predictors for Pretherapy Characterization and Therapy Selection

Few studies in the literature investigated the usefulness of a detailed knowledge of patients’ motor capabilities for a pretherapy detailed assessment. Such a feature would give the clinician the capability of selecting a sequence of interventions that proved in the past to be effective on that specific group of patients. Muscular synergies have relevant potential under this point of view. They are extracted directly from the stimulation given by the nerves to the muscles on the patient and are not the result of tests, clinical scales or kinematic outcome variables. Furthermore, synergies approach can be tuned by selecting, either (1) synthetic approaches, trying to reduce the number of clusters and classifications to synthetize groups and interventions (especially useful on a limited sample, like in this study); (2) analytic approaches, detailing many more clusters for capturing even minor motor differences (which, on a wide statistical sample, may be the preferable approach). Interestingly, the synthetic approach could be useful to identify macrodivisions, such as deciding if a patient is more suitable for a specific therapy approach in a restricted range of choices. On the contrary, the analytic approach might be chosen to specify the more suitable detail on refined customization.

To authors’ knowledge, no studies, either in acute or in chronic stage stroke patients, have explored the possibility to characterize the patient and the therapy merging two or more methods. Especially in the upper limb rehabilitation field, where less results are usually obtained in comparison to the lower limb, an integrated approach which combines more variables and different levels of evaluation, from anatomical to functional, could be the solution for a real therapy customization according to the predicted outcomes. Muscle synergy characterization could play a valuable role in prediction of prognostic outcomes, also in association with previous techniques. In frontal reaching movements, patients with prevalence of shoulder synergies, dominated by elevator muscles (such as Da, middle, and Tr), might be candidates for motor recovery. Prevalence of elbow activity is instead a bad prognostic index, indicating the impossibility to perform the gesture. Such patients would need antigravitary support to perform reaching movements. For the reasons explained, synergies could be integrated in a multifactorial assessment to better estimate the residual potential of the patient and consequently suggest the best rehabilitation path available. Moreover, synergies extraction could be useful for a real-time EMG-based robot interaction for an improved compliance during a robotic rehabilitation protocol. Potential application includes pathology-specific synergy characterization can be useful also for an efficient EMG-based robotic control: the real-time patient’s EMGs could be mapped in the synergy space, with real time estimation of the time-varying activation coefficients (Lunardini et al., 2016). Further investigations on muscle synergies measured before and after a training period could demonstrate if, beyond synergy modification which has been already established, there could also be a cluster modification or cluster transfer between patients, defining the validity of the approach for prediction of outcome variations.

To choose the most suitable approach of rehabilitation links to the concept of predicting the therapy outcome. In fact, selecting a therapy implicitly assumes that the clinician specifies motor functions to be trained and expected results.

A class of predictors includes imaging assessment like transcranial magnetic stimulation or magnetic resonance imaging to verify the integrity of the corticospinal tract and brain (Puig et al., 2011). Both instruments can give more reliable information about the general condition of the patient are far more expensive, time-consuming and have some contraindications. Muscle synergies demonstrate good reliability in describing the EMG pattern organization but they have never been exploited as outcome predictors.

The cited techniques mostly focus on acute stroke to understand the residual potential of recovery and consequently quickly address to specific rehabilitation paths. Less methods take into account the chronic phase of the pathology, despite some improvements can be achieved. Some experiments have been conducted with EEG signals that show differences on potential recovery of chronic subjects. These results can be exploited to understand who needs further exercise therapy (Trujillo et al., 2017). Also the corticospinal tract status seemed to correlate with motor skill even in the chronic stage (Schaechter et al., 2009).

## Conclusion

In this article, the problem of patients’ characterization before therapy is addressed and discussed in the framework of muscular synergies. A clustering technique is proposed, along with clinical profiling, for patients’ characterization, based on muscular coactivation patterns. Such procedure identified a trade-off solution of five clusters on a population sample of 22 poststroke subjects. Each cluster was characterized by specific compensatory strategies due to impairment. Interestingly, the muscular synergies profiling does not match precisely the evaluation provided by clinical scales, suggesting that the evaluation provided by standard tools should be integrated for complete assessment and patients’ characterization. Further studies will investigate the generalizing power of the method and of the identified groups, in a pre–post rehabilitation trial.

Furthermore, muscle synergies extraction and clustering might be used also to describe critically the effects of a therapy. Results could be observed by groups as a whole, or observing single patients. The first result that can be observed is related to the number of clustered needed to “explain” patients’ motor behavior in respect to the beginning of the therapy. A change in the number of clusters might indicate convergence toward specific patterns (which may be promoted by the rehabilitation approach) or *vice versa*. Even dispersion inside clusters may be indicators of the goodness of the clustering and its evolution in time. Single patients may present transitions from a cluster region to another. Such a result can be of great interest in evaluating the modifications of patients’ motor behavior and to understand which patients may achieve higher benefits due to the therapy.

Further studies will test the method proposed in this article for the assessment of the effect of robotic therapy approach to upper-limb rehabilitation. Modifications of clusters and shifting from one clusters to another will be considered as valuable insights for assessing the efficacy of the therapy.

## Ethics Statement

This study was carried out in accordance with the recommendations of local Ethics Committee at Lecco Manzoni Hospital with written informed consent from all subjects. All subjects gave written informed consent in accordance with the Declaration of Helsinki. The protocol was approved by the local Ethics Committee at Lecco Manzoni Hospital.

## Author Contributions

AS designed the experiment, wrote the software for synergies extraction, performed the experimental campaign, elaborated the data, and wrote the article. AC performed the experimental campaign and participated to data analysis and interpretation. MM was responsible for the research project that funded the work. He revised the article and participated to data analysis and discussion. LT was the head of the CNR research group. He revised the article and participated to data analysis and discussion. FM is the head of the Villa Beretta Hospital and participated to the conception of the evaluation system, and gave clinical interpretation to the data.

## Conflict of Interest Statement

The authors declare that the research was conducted in the absence of any commercial or financial relationships that could be construed as a potential conflict of interest.
